# Health care for all: effective, community supported, healthcare with innovative use of telemedicine technology

**DOI:** 10.1186/s40545-018-0130-5

**Published:** 2018-02-01

**Authors:** Tariq Kazim Shah, Tasneem Tariq, Roger Phillips, Steve Davison, Adam Hoare, Syed Shahzad Hasan, Zaheer-Ud-Din Babar

**Affiliations:** 1 Health Care 4 All International, Willow House 107 All Alone Road Bradford, Bradford, West Yorkshire BD10 8TR UK; 2 Department of Pharmacy, School of Applied Sciences, University of Huddersfield, Huddersfield, HD1 3DH UK; 3 Bayswater Institute, 9 Orme Court, London, W2 4RL UK; 4 School of Pharmacy, Faculty of Medical and Health Sciences, University of Auckland, Auckland, Private Mail Bag 92019 New Zealand

**Keywords:** Primary, Healthcare, Remote rural communities, Telecommunication, Technology, Innovation, Telemedicine

## Abstract

Almost half of the world’s total population reside in rural and remote areas and a large number of these people remain deprived of most basic facilities like healthcare and education. It is deemed impossible for government with scarce resources in developing countries to open and run a health facility in every remote community using conventional means. One increasingly popular unconventional mean is the use of existing technology to improve exchange of medical information for the purpose of improving health of underprivileged communities. Telemedicine implies the use of information and communication technology to provide health care remotely from a distance. With the induction of telemedicine, patients who live in rural and remote areas can have increased access to medical services. In many developing countries, use of telemedicine however has been limited mainly to teleconferencing between primary and secondary/tertiary care facilities for diagnosis and management of patients. This system still requires patients from remote communities to travel, often long and arduous journeys to the centre where telecom and medical facilities are available. Health Care 4 All International, a not for profit registered charity is providing primary care to patients by taking telemedicine into their homes in remote communities, thus obviating the need and hardships of travel for patient.

## Introduction

In 2015, United Nations published a report about the achievements of the Millennium Development Goals (MDGs) for the past 15 years. Although ‘dramatic progress’ has been made and all MDG’s influence health [1, 2], there still exists inequalities in healthcare provision between the high and low income countries. There are also stark differences between the urban and rural population. United Nations Department of Economics & Social Affairs highlighted that there is about a 50:50 divide between the urban and rural population yet only 26% of world’s medical doctors and 32% of the world’s nurses provide healthcare services in the rural areas [3–5]. Almost all (99%) of both child and maternal mortality occur in the low and middle-income countries and are highest in the rural communities [6, 7].

Information and Communication technology (ICT) has shown huge potential to overcome public health challenges and in this context, telemedicine can play an important role. Telemedicine is the use of ICT to provide clinical health care from a distance. It has been used to overcome distance barriers and to improve access to medical services. Access, equity, quality, and cost-effectiveness are key issues facing healthcare in both developed and less economically developed countries [8].

Conventional telemedicine requires a fast and reliable telecommunication network, and both the service user and provider to have telecommunication equipment and the knowledge to use them. This is very challenging in the developing countries, particularly in the remote rural communities. Cost also prohibits the use of telemedicine in its conventional form. In this context, a new model is being developed to promote safe and cost-effective use of these resources in a low-income country setting. In this editorial, we discuss about the use and functioning of this model.

Health Care 4 All International (HC4AI) is a not for profit registered charity that has innovated and modified Telemedicine to suit the environment prevailing in the selected area of deployment. We call this ‘Novel Hybrid System of Telemedicine’ (NHST). HC4AI has been providing healthcare successfully for over three years in the villages of Kashmir in Pakistan. In this editorial, we present how NHST works and our experience.

## HC4AI solution: Novel Hybrid System of Telemedicine

We selected four men and four women from these villages with some paramedical background, trained them in basic use of technology and updated on routine and emergency medical practice. They were then provided with computer tablets downloaded with our own developed Electronic Health Record (EHR) software and located them in a central place (Monitoring Centre) from where they can reach any household in the village when called. These ‘Community Health Workers’ (CHW) became the mouthpiece for the community-an interface between the patient in their homes and the doctor located remotely. They provided a round the clock service. They also carry a ‘tool bag’ containing diagnostic equipment, first aid kit, set of routinely used medicines and a portable telecommunication antenna.

As no suitable bandwidth was available in the area, we established our own telecommunication network with the help of Mirpur University of Science and Technology [9] Every individual in the community is registered with a unique ID number, entered on EHR with medical history and provided with a single telephone number to call whenever they need medical help. We also acquired two ambulances equipped with a communicating antenna, and two salaried doctors, a male and a female, to work 24 h on-call schedule in tandem. Specialists who agreed to provide service voluntarily or for a fees per case basis were also appointed covering the mainline specialities of medicine, surgery, paediatrics, obstetrics & gynaecology and orthopaedics. The project was started in 2014 as a pilot study covering a population of 1500 spread in eight small villages. However with rapid success, it was converted into a full service in early 2015. It has since gradually expanded into surrounding villages at minimal extra cost and now covers a population of nearly 50,000 inhabitants spread over in more than thirty villages.

## How does the system work

When a call is received at the monitoring centre the on-call CHW team goes to the caller in the ambulance, if the terrain is inaccessible to the ambulance, the CHW have to walk. After noting the history and examination on the EHR, the CHW contacts the doctor via video/audio link, who can interview the patient directly if required. The doctor then makes the diagnosis and suggests treatment; the CHW dispenses the medicine to the patient. More serious patients are evacuated in the same ambulance to the base and if specialist care is required, then the patient is transferred to the specialist centre. Every step of the patients’ journey is recorded on the EHR as well as on the central database. An evaluation of the service, (Pre- and Post-NHST Intervention) of 350 households shows extremely positive experience especially in the provision of health services, access to medical care and advice to the family.

## Concluding remarks

We believe that the success of the model depends on building on a local approach. The cultural and religious beliefs need to be understood and should be kept in perspective when designing these services. Also, the success depends on having a strong base in the community. This is also vital in the context, to identify the needs, acceptance and ownership of these services. Another take home lesson is that as much as possible, the workforce should be selected from these communities.
